# The role of diagnostic technologies to measure progress toward WHO 2030 targets for soil-transmitted helminth control programs

**DOI:** 10.1371/journal.pntd.0009422

**Published:** 2021-06-03

**Authors:** Lieven J. Stuyver, Bruno Levecke

**Affiliations:** 1 Global Public Health R&D, Janssen Pharmaceutica NV, Beerse, Belgium; 2 Department of Virology, Parasitology and Immunology, Ghent University, Merelbeke, Belgium; Liverpool School of Tropical Medicine, UNITED KINGDOM

## Background

Infections with soil-transmitted helminths (STHs) occurs throughout the developing world and are caused by 5 main species commonly known as roundworms (*Ascaris lumbricoides*), whipworms (*Trichuris trichiura*), hookworms (*Ancylostoma duodenale* and *Necator americanus*), and threadworm (*Strongyloides*). Recently, the World Health Organization (WHO) published its 2030 roadmap for STH preventive chemotherapy (PC) control programs [1]. In this roadmap, a total of 6 ambitious targets were identified, each with their corresponding milestones. The first 2 targets are to achieve and maintain elimination of STH-attributable (excluding *Strongyloides*) morbidity in pre-school age children (pre-SAC) and school-age children (SAC) by 2030 (**Target #1**) and to reduce the number of tablets needed in PC (**Target #2**). **Target #3** aims increase domestic financial to support PC, whereas **Targets #4** and **#5** are aiming to establish an efficient control program specific for woman of reproductive age and strongyloidiasis in SAC, respectively. Finally, **Target #6** aims to achieve universal access to basic sanitation and hygiene in STH-endemic areas. In the present viewpoint, we will reflect on the diagnostic technologies to measure progress toward **Target #1** and **Target #2** only. There, cost-effective diagnostics are a prerequisite to measure progress toward the set milestones. For **Target #1**, the milestone is the number of countries that have successfully reduced the prevalence of moderate and heavy intensity (M&HI) infections in children to less than 2% (2023: 70 countries; 2025: 90 countries; and 2030: 98 countries), whereas for **Target #2**, the milestone is the percentage reduction in number of anthelmintic tablets needed to deworm pre-SAC and SAC (2023: 20%; 2025: 30%; and 2030: 50%). For the latter target, a performant data reporting system feeds a decision tree to scale down the frequency of PC programs (and hence the number of anthelmintic tablets) based on prevalence of any STH infections. For the other targets, milestone indicators are in one way or another a coverage metric (**Targets #4 to #6**).

In the 2030 WHO roadmap, the Kato-Katz (KK) thick stool smear remains the recommended diagnostic standard to detect and quantify the intensity of STH infections, although reference to other yet unspecified quantitative diagnostics is made. The KK method is not considered as the method of choice to detect *Strongyloides* infections; therefore, we will not further discuss strongyloidiosis in the context of achieving **Targets #1** and **#2**. A recent diagnostic gap and priority assessment concluded that current diagnostic technologies are adequate—providing some minor modifications—for mapping STH infections and initiating PC programs, but not adequate at all when PC programs matures toward stopping decisions and post-PC surveillance [2]. The latter is especially true when the proposed diagnostic specifications move away from stool and are prioritizing non-stool-based technology developments (e.g., for urine and serum samples).

In the present viewpoint, we first identified the key diagnostic attributes for technologies to measure progress toward WHO **Targets #1** and **#2** for 2030. Subsequently, we verified which existing technologies can address these attributes and how they compare to the currently recommended KK method when applied in a programmatic setting. Finally, we identified some opportunities to improve existing diagnostic technologies.

### Key diagnostic attributes needed to measure program progress

When **Target #1** is interpreted from the diagnostic perspective, technologies will need to meet the following specifications: (i) provide information on STH-attributable morbidity; (ii) generate quantitative readout (iii) for each of the 4 STH species separately (multiplexing); (iv) have a clinical sensitivity of at least 95% for M&HI infections but similar to single KK for low intensity infections; and (v) clinical specificity equal or superior of a single KK in individuals with M&HI infections [2]. In case of non-stool-based testing, the clinical sensitivity should be superior to microscopy-based tests and clinical specificity equal or superior to quantitative polymerase chain reaction (qPCR)-based measurements [2]. These sensitivity and specificity parameters were ill-defined as guidance for new test development, and obviously open for further refinement. Furthermore, additional insights on sensitivity and specificity requirements for low prevalence and elimination settings detailed the importance of test specificity over sensitivity [3]. Concerning the STH morbidity attribute, it is impossible to measure the exact number of worms in a host, hence the relationship between the number of worms and morbidity remains elusive [4]. However, there is a relationship between the number of worms and the number of eggs in stool [5], although this relationship has many weaknesses [6]. In absence of any better morbidity measurement, quantifying fecal egg counts (FECs) per gram stool (eggs per gram stool (EPG)) remains the best proxy, implying stool-based testing.

For **Target #2**, the diagnostic technologies should be fully integrated in the program decision process, including built-in data analysis and reporting for streamlined communication of results and connection to national data servers to follow up progress toward national program targets and to estimate the number of anthelmintic tablets needed for the upcoming year. The **Target #2** values for diagnostic performance parameters are essentially identical to **Target #1**, yet now apply for infections of any intensity.

In addition, there are a number of general attributes—the so-called Affordable, Sensitive, Specific, User-friendly, Rapid and robust, Equipment-free and Deliverable to end-users (ASSURED) criteria—that address the poor resource setting in which current STH programs traditionally operate [7]. ASSURED criteria are not limited to **Targets #1** and **4**, but also for **Target #2** (number of drugs will be dependent on the availability of diagnostic technology that is guiding the decision process with high accuracy data).

### Landscape analysis of diagnostic technologies for STH in a programmatic setting

**Table 1** provides an overview of the technologies/biomarkers that have been evaluated for the detection and quantification of human STH infections. Although some of the stool-based technologies have successfully moved toward field testing, the identification and evaluation of biomarkers in non-stool samples have been rather sobering [8]. A proof of principle of 2-methyl-pentanoyl-carnitine (2-MPC) as metabolite biomarker in urine and serum/plasma was evidenced for *A*. *lumbricoides*, but despite intense research efforts, not for the other species [9]. Other research groups are working with success on biomarker discoveries for *Schistosoma* spp. [10], and similar approaches might lead to new candidates for STH as well. The latter study also indicates that there is a considerable knowledge gap between STH and Schistosomiasis (SCH) when it concerns diagnostic biomarkers, in which SCH is leading the field with years of research and development. For STH and considering these biomarker discovery challenges and the timelines and costs associated with test development, we argue that the much desired non-stool-based transformational technology is out of scope for the 2030 WHO STH roadmap. The mentioned observations should not impact the high expectation of new biomarker-based diagnostics beyond the 2030 roadmap.

**Table 1:** An overview of the technologies or biomarkers that have been evaluated for human STH.

Sample type	Biomarker	STH	Name/technology	Status	References
Stool	Eggs	All	FLOTAC	Field tested	[21]
			Mini-FLOTAC	Field tested	[15,18]
			FECPAK^G2^	Field tested	[15,22]
			Lab-on-disk	Proof of principle	[17]
		All	Imaging: HEAD	Proof of principle	[23]
		All	Imaging: KANKANET	Proof of principle	[16]
	DNA of eggs/worms	All	qPCR	Field tested	[15]
			LAMP	Proof of principle	[24]
	ABA-1 coproantigen	*Ascaris*	ELISA	Proof of principle	[11]
Urine	2-MPC	*Ascaris*	LC–MS	Proof of principle	[9]
Serum/plasma	Antibodies against worm hemoglobin	*Ascaris*	ELISA	Proof of principle	[25]
	Antibodies against third larval lung stage	*Ascaris*	ELISA	Proof of principle	[26]
	2-MPC	*Ascaris*	LC–MS	Proof of principle	[9]

2-MPC, 2-methyl-pentanoyl-carnitin; ELISA, enzyme-linked immunosorbent assay; LC–MS, liquid chromatography–mass spectrometry; qPCR, quantitative polymerase chain reaction; STH, soil-transmitted helminth.

Among the innovative stool-based technologies, copro-antigen detection (presented in a multiplex lateral flow assay) is one possible way forward. However, our experience with the *A*. *lumbricoides* copro-antigen ABA-1 [11] points toward the same complexity as for the non-stool-based approaches, namely that (i) stool biomarkers are not yet identified for all STHs; (ii) multiplexing is possible only after (iii) sensitivity; and (iv) specificity for each biomarker and for each infection is fully optimized. Moreover, (v) the relationship between antigens and morbidity is yet to be determined (if possible at all); and (vi) additional processing steps might be required (bead beating) which affect the user-friendliness. Therefore, it is unlikely that such copro-antigen technologies will become available within the time frame of the 2030 roadmap.

In **Table 2**, we verified to which extent the remaining stool-based technologies that have moved toward field testing align with the key diagnostic attributes. For **Target #1,** the current standard KK is set as the reference method, meeting the required sensitivity and specificity to detect and classify M&HI infections. Comparing to the reference method, other technologies do not achieve a reliable classification of infection intensities across the different STH species [12]. For **Target #2**, only qPCR technology achieves the required data accuracy. The biggest hurdle for a general adoption of the qPCR technology is the complexity of the assay, the cost of materials, the extended time to result (mainly due to the labor-intensive DNA extraction), and the lack of standardization [13]. It therefore does not come as a surprise that most STH qPCR studies were conducted on stool samples collected in endemic countries but shipped to specialized laboratories. Implementing qPCR methods in endemic countries is theoretically possible, but only after a technology, infrastructure, and financial support upgrade and providing training of local staff.

**Table 2:** Matching current stool-based technologies with the diagnostic attributes to measure or make progress toward 2030 targets.

	KK (single)	Mini-FLOTAC	FECPAK^G2^	qPCR
Target-specific attributes				
*Target 1* [2,12,27]				
Morbidity	FECs as a proxy			Amount of DNA of eggs or worms as a proxy
Infection intensity thresholds	WHO endorsed	Proposed, but not endorsed by WHO		
Quantitative readout	Eggs per gram of stool			No consensus on a universal unit
Multiplex	All STH, excluding differentiation of hookworm spp. and *Strongyloides*			All STH including differentiation of hookworm spp. and *Strongyloides*
Clinical sensitivity	Reference method	*Ascaris*: 94.7%	*Ascaris*: 91.0%	*Ascaris*: 83.5%
		*Trichuris*: 93.5%	*Trichuris*: 78.6%	*Trichuris*: 87.2%
		Hookworms: 87.9%	Hookworms: 87.9%	Hookworms: 78.8%
Clinical specificity	Reference method	*Ascaris*: 92.0%	*Ascaris*: 84.1%	*Ascaris*: 87.8%
		*Trichuris*: 90.6%	*Trichuris*: 79.6%	*Trichuris*: 75.0%
		Hookworms: 90.8%	Hookworms: 86.4%	Hookworms: 88.5%
***Target 2*** [15,28]				
Clinical sensitivity: any intensity	*Ascaris*: 71.9%	*Ascaris*: 63.3%	*Ascaris*: 58.9%	*Ascaris*: 90.0%
	*Trichuris*: 88.1%	*Trichuris*: 91.5%	*Trichuris*: 59.8%	*Trichuris*: 94.7%
	Hookworms: 72.6%	Hookworms: 73.9%	Hookworms: 52.4%	Hookworms: 91.9%
Clinical sensitivity: low intensity	*Ascaris*: 55.6%	*Ascaris*: 42.1%	*Ascaris*: 36.8%	*Ascaris*: 86.2%
	*Trichuris*: 79.6%	*Trichuris*: 85.6%	*Trichuris*: 37.5%	*Trichuris*: 91.0%
	Hookworms: 69.4%	Hookworms: 70.8%	Hookworms: 47.5%	Hookworms: 91.0%
Clinical sensitivity: M&HI	⩾95.0% for all STH	⩾95.0% for all STH	⩾95.0% for *Ascaris* only	⩾95.0% for all STH
Clinical specificity	Assumed to be ⩾95.0%			Assumed to be 100%
**General attributes**				
***ASSURED***				
Time to result	412 s	620 s	758 s	Not yet evaluated
Material cost	US$1.38	US$1.52	US$1.96	To be determined
Supplier	Multiple	Single	Single	Multiple
Hardware	Microscope/power supply	Microscope/power supply/KUBIC	Computer, Micro-I/power supply	Extraction/amplification equipment; power supply
Reagents accessible in STH-endemic countries	Easily	Easily	Easily	Complicated, cold chain is needed
***Integration into program decision-making***				
Data entry, data analysis, and reporting	Manual		Automated, proof of principle in veterinary parasitology	Manual (depending on laboratory information management system)

FEC, fecal egg count; KK, Kato-Katz; M&HI, moderate and heavy intensity; qPCR, quantitative polymerase chain reaction; STH, soil-transmitted helminth; WHO, World Health Organization.

Based on this landscape analysis, we conclude that the KK reference method is here to stay for at least another decade and will likely be the only general instrument to inform WHO STH 2030 roadmap.

### Low hanging fruit to improve the current reference method

There is a lot of controversy around the performance of the KK procedure, and often the lack of sensitivity, reproducibility, and error-prone manual readout are considered major shortcomings [14]. Surprisingly enough, specificity of KK has not been seen as a drawback, while it is exactly that performance requirement that is crucial in low prevalence and elimination settings [3]. Contrary to these observations, some reports demonstrate accurate performance [15]. This controversy suggests that the KK procedure is in principle a valid method, but improvements in the readout and reporting procedure could alleviate the shortcomings. The integration of egg detection and readout technologies have been prototyped in, e.g., the FECPAK^G2^, KANKANET, spin-disc platforms, and mini-FLOTAC KUBIC [15–18]. However, the field experience of these technologies provide lessons around complexity and turnaround time as compared to the reference method (**Table 2**).

We envision that improvements to the traditional KK method might come from automation in data collection, analysis, and reporting, which are known to be the most laborious and time-demanding steps. Automation is not aiming to solve the methodological drawback of the KK procedure, but only aiming to improve the accuracy of the readout and reducing the operational costs both to process samples and to write final reports (which, in turn, could be reinvested in expanding the sampling area, and hence improve the program decision-making).

Developments into the field of digital pathology linked to artificial intelligence (AI) might be applied to KK, provided they can be made cost comparable to the current manual procedure (“affordable digital pathology”). The principle of an automated KK concept has been demonstrated (proof of technical feasibility is available) [16,18], and the development only depends on the availability of image databases and the integration of engineering activities to mitigate the shortcomings of the error-prone microscopic manual readout and reporting.

## Conclusions

Strengthening diagnostic capacity is often being put forward as a top priority in the field of neglected tropical diseases (NTDs), and STH in particular. Yet, overall investments in this area has thus far been limited, representing about 5% of R&D investments for NTDs [19]. The raging Coronavirus Disease 2019 (COVID-19) pandemic is even further limiting or redirecting the scarce NTD diagnostics funding [20].

Prioritizing non-stool-based diagnostic platforms for the STH 2030 targets is extremely ambitious, and in the long run, may turn into an “appeal to future discovery fallacy.” The traditional KK method is currently fit for purpose. With a proper focus and funding for automation and AI-driven readout, the introduction of a KK-based transformational technology that can fully support WHO 2030 STH roadmap is expected.
